# Impact of COVID-19 pandemic on outpatient visit volume in cancer patients: Results of COMETA multicenter retrospective observational study

**DOI:** 10.3389/fpubh.2023.1077103

**Published:** 2023-02-14

**Authors:** Vincenza Frisardi, Oronzo Brunetti, Vincenza Abbinante, Marco Ardigò, Giuseppina Caolo, Annunziata Di Turi, Alessandra Torsello, Christian Napoli, Rita Mancini, Valeria Belleudi, Antonio Addis, Ornella Di Bella, Gennaro Ciliberto, Antonino Neri, Romina Corsini, Patrizia Ruggieri, Chiara Pollorsi, Nicola Silvestris

**Affiliations:** ^1^Department of Neuromotor Diseases, Geriatric Unit, AUSL IRCCS of Reggio Emilia, Reggio Emilia, Italy; ^2^Istituto Tumori “Giovanni Paolo II” of Bari, IRCCS, Bari, Italy; ^3^San Gallicano Dermatological Institute IRCCS, Rome, Italy; ^4^National Cancer Institute IRCCS Regina Elena, Rome, Italy; ^5^Sapienza University of Rome, Saint'Andrea Hospital, Rome, Lazio, Italy; ^6^Department of Epidemiology, Lazio Regional Health Service, Rome, Italy; ^7^Scientific Directorate, AUSL-IRCCS of Reggio Emilia, Reggio Emilia, Italy; ^8^Medical Oncology Unit, Department of Human Pathology “G. Barresi”, University of Messina, Messina, Italy

**Keywords:** COVID-19, healthcare research, cancer care facilities, outpatients, clinic activity, oncology service

## Abstract

**Objective:**

To evaluate the impact of the COVID-19 pandemic on first and follow-up visits for cancer outpatients.

**Methods:**

This is a multicenter retrospective observational study involving three Comprehensive Cancer Care Centers (CCCCs): IFO, including IRE and ISG in Rome, AUSL-IRCCS of Reggio Emilia, and IRCCS Giovanni Paolo II in Bari) and one oncology department in a Community Hospital (Saint'Andrea Hospital, Rome). From 1 January 2020 and 31 December 2021, we evaluated the volume of outpatient consultations (first visits and follow-up), comparing them with the pre-pandemic year (2019). Results were analyzed by quarter according to the Rt (real-time indicator used to assess the evolution of the pandemic). IFO and IRCCS Giovanni Paolo II were “COVID-free” while AUSL-IRCCS RE was a “COVID-mixed” Institute. Depending on the Rt, Sain't Andrea Hospital experienced a “swinging” organizational pathway (COVID-free/ COVID-mixed).

**Results:**

Regarding the “first appointments”, in 2020 the healthcare facilities operating in the North and Center of Italy showed a downward trend. In 2021, only AUSL-IRCCS RE showed an upward trend. Regarding the “follow-up”, only AUSL IRCCS RE showed a slight up-trend in 2020. In 2021, IFO showed an increasing trend, while S. Andrea Hospital showed a negative plateau. Surprisingly, IRCCS Giovanni Paolo II in Bari showed an uptrend for both first appointment and follow-ups during pandemic and late pandemic except for the fourth quarter of 2021.

**Conclusions:**

During the first pandemic wave, no significant difference was observed amongst COVID-free and COVID-mixed Institutes and between CCCCs and a Community Hospital. In 2021 (“late pandemic year”), it has been more convenient to organize COVID-mixed pathway in the CCCCs rather than to keep the Institutions COVID-free. A swinging modality in the Community Hospital did not offer positive results in term of visit volumes. Our study about the impact of COVID-19 pandemic on visit volume in cancer outpatients may help health systems to optimize the post-pandemic use of resources and improve healthcare policies.

## 1. Introduction

The COVID-19 pandemic was an unprecedented experience for our modern health systems and is a landmark for future national and international action plans and programs, especially for vulnerable people with cancer. It is well known that malignancies and cancer therapies lead to systemic immunosuppression, which exposes these patients to infection more than others, especially severe COVID-19. In addition, the greatest concern for cancer patients is their inability to receive essential medical services due to the spread of the pandemic (1).

Comprehensive Cancer Care Centers (CCCCs) have been established worldwide to improve care pathways for cancer patients.^1^ In response to COVID-19, national health policies and local organizational decisions forced oncology centers to reorganize their healthcare performance to ensure access to medical services. Italian healthcare institutions went through three distinct phases during the first year of the pandemic in the fight against COVID-19. The first began at the end of February 2020, when most infections occurred in the northern regions (Lombardy, Piedmont, Emilia Romagna, and Veneto). The Italian government imposed a strict lockdown until May. The second phase, in which the contagion curve began to stabilize below the threshold value, lasted from June to the end of September. The third phase started in October 2020 – corresponding to the second wave of infection, which had a much larger scale than the first (2, 3). In Italy, some healthcare facilities admitted patients regardless of whether their SARS-CoV2 test was positive or negative, while other hospitals where COVID-free and required a negative test for admission.

Numerous findings have been published about COVID-19 and continuity of care for cancer patients (4–6). A Spanish study found that the pandemic had an early impact on routine practice in hematology and medical oncology, with a 20.8 and 21.2% decrease in average number of new visits, respectively (a surrogate for newly diagnosed cases) (7). The international scientific guideline and national policies have led oncologists to act according to the local task force; in particular, follow-up and screening have been postponed, and it has been recommended to transfer inpatients to the outpatient setting when possible (8). Nevertheless, there are no data on the different adopted strategies and changes in outpatient appointments during the COVID-19 pandemic. The purpose of this study is to analyze the extent of medical visits for outpatient cancer patients (both for the first appointment and for follow-up) during the two-year pandemic 2020–2021 and to better characterize the trend and the impact of COVID-19 in the care of outpatient cancer patients.

## 2. Methods

**Figure 1:**
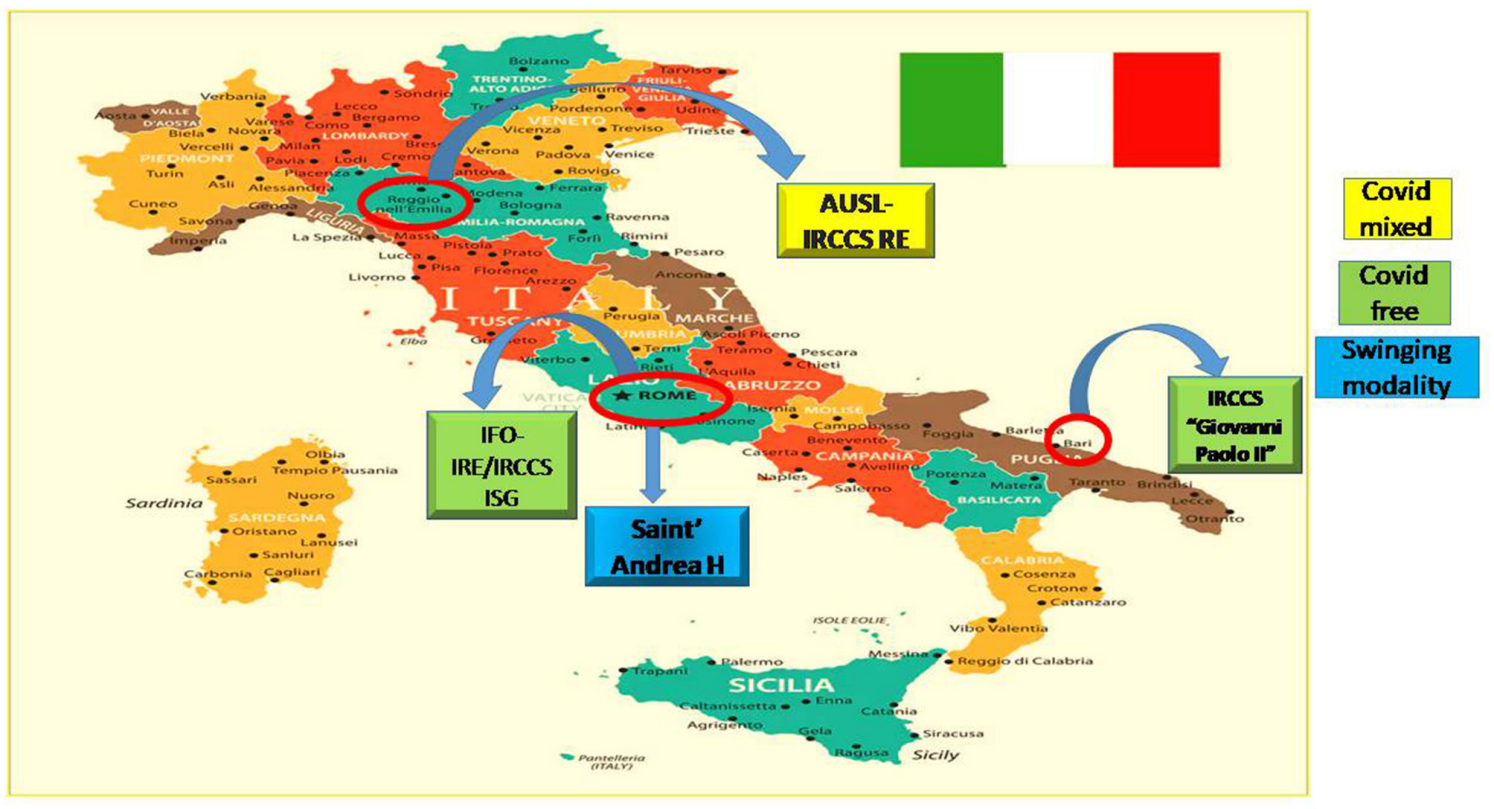
Map of the clinical centers involved in the COMETA project. AUSL IRCCS (Reggio Emilia), IRCCS IFO/IRE (Rome), and IRCCS “Giovanni Paolo II (Bari) are CCC (Comprehensive Cancer Care) centers. Saint' Andrea Hospital is a University Hospital Center based in Rome.

This study is embedded in an Italian project, namely the COMETA project, which involved three CCCCs and an oncology department in a Community Hospital (Saint'Andrea Hospital). The CCCCs are (1) The Istituti Fisioterapici Ospitalieri (IFO) that comprised of two institutes [the Regina Elena National Cancer Institute (IRE) and the Dermatological Institute S. Gallicano (ISG)], (2) AUSL-IRCCS RE and 3) IRCCS “Giovanni Paolo II”. These health care facilities are located in three different geographical areas: Reggio Emilia (Emilia-Romagna, northern Italy, population 168,862), Rome (Lazio, central Italy, population 2,763,804), and Bari (Puglia, southern Italy, population 317,017) (Figure 1). Our study represents one of the objectives of the multicenter COMETA project, the aim of which was to develop approaches and metrics to assess the impact of COVID-19 and improve clinical outcomes of patients with frailty (9). In particular, we investigated how the management of the pandemic and patient safety affected the volume of outpatient consultations. To this purpose, we considered (i) the number of the first appointments and (ii) the number of follow-up appointments. A consensus decision-making process was conducted among the experts involved in the COMETA project and used to define these indicators. The main characteristics of the key performance indicators (KPIs) are listed in Table 1 [for more details, see the protocol of the COMETA project Aim 2 (9)].

**Table 1:** Criteria for the choice of indicators.

**Characteristics**	**Agreement**	**Criterion's importance**
Extractable data by Direction of Health (avoiding the extra burden for clinicians)	98%	Relevant
Easily accessibile	100%	Very important
Easily comparable across the involved centers	100%	Very important
Measurable (quantitative)	100%	Important
Representative of the corporate performance indicators (access and service provided)	100%	Very important

We selected data according to International Classification of Diseases-10 codes and statewide cancer exception codes (neoplasm exception code: 048) by matching results from administrative data streams associated with the Operations Registry and the Unified Regional Accounting Center. We included all types of neoplasms (hematologic and solid tumors) and obtained data on the volume of first and follow-up appointments. Data from the official flow of information of outpatient specialist services, which ensures a uniform and homogeneous method of data collection, were used to equalize “outpatients non-attendance”. We selected visit data with STATUS “Accepted/Completed” and then divided into First Visit and Follow-up visit.

**Figure 2:**
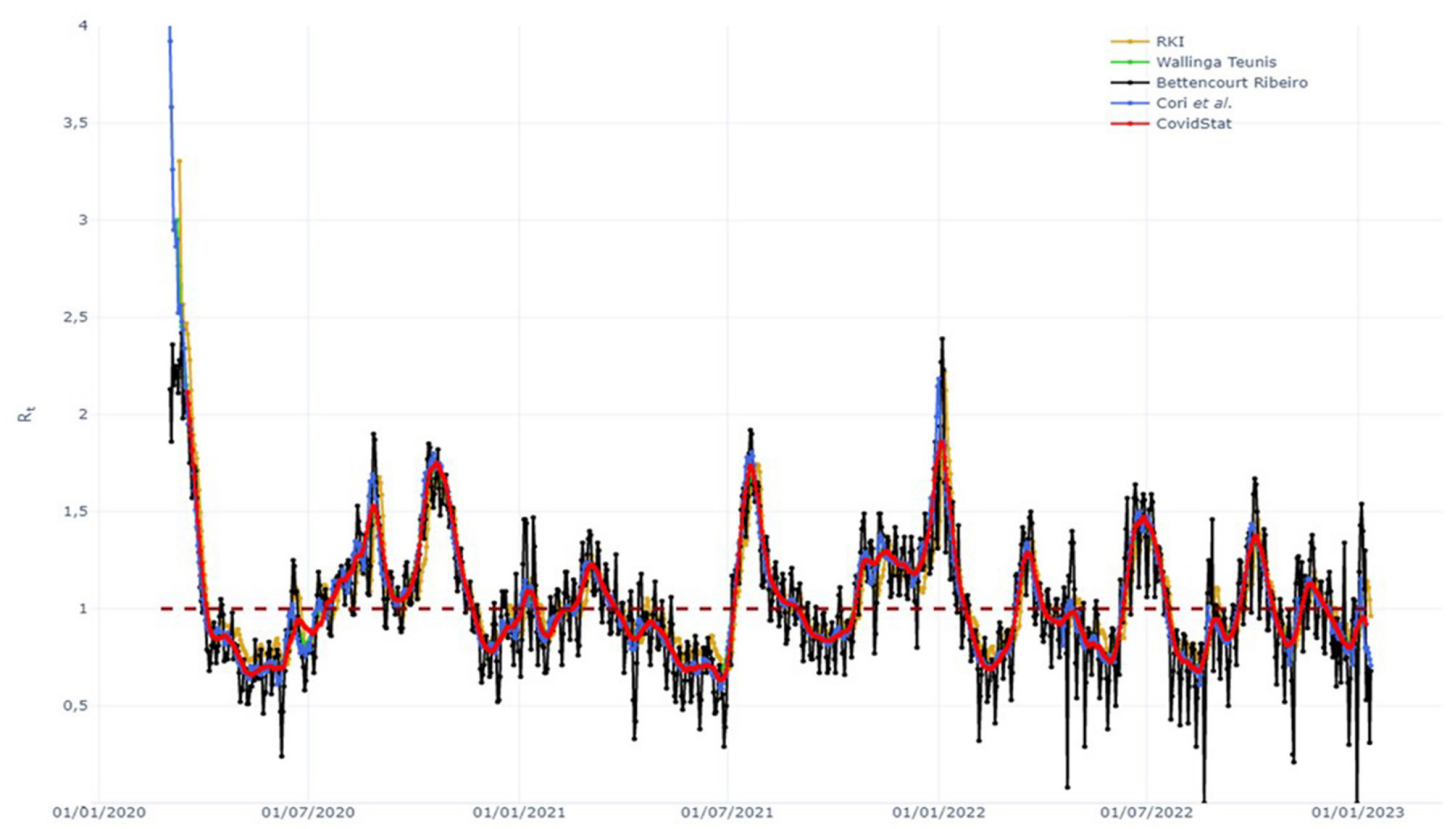
Example of COVIDStat Rt trend report. National framework adapted from the website: https://covid19.infn.it/sommario/rt.html.

We considered the period from January 1, 2019 to December 31, 2021, and defined the 3 years as “pre-pandemic (*pp*), pandemic (*p*), and late pandemic (*lp*).” In addition, we subdivided each year by quarter based on (i) the presumed role of seasonality in the spread of the COVID- 19 pandemic (10) and (ii) the availability of the quarterly grouped administrative data flow. Data were compared with the year prior to the pandemic, and differences were discussed based on the Rt value (the effective reproduction number) of each region. Rt is a real-time indicator used to assess the evolution of the pandemic, design containment measures, and monitor their effectiveness at a contagion rate. Figure 2 shows how the Rt value for a single region was extrapolated data from the INFN Open Access Repository^2^ selecting the COVIDStat method to calculate Rt trend (see text footnote ^2^). In this website, we were able to visualize Rt data by region or for the whole country as an average in the specific time period. Daily Rt values were aggregated to weekly averages and signed for quarters. We considered “1” as the epidemic threshold (Figure 2).

For our analyses, we used anonymized data; these data are routinely collected for administrative purposes and converge in the national information system of the Italian Ministry of Health. Because the use of these data is mandatory to track healthcare performance for the Italian health system, the written consent of patients is not required by the current regulation. Nevertheless, the ethics committee of the promoter and each participating institution approved the COMETA project. Data were processed in accordance with the relevant data protection regulations. The study followed the guidelines of STrengthening the Reporting of OBservational studies in Epidemiology (STROBE) (11). Data analysis was performed using SPSS, version 22 (Statistical Package for Social Science; SPSS Inc. Chicago, IL, USA). Categorical variables are presented as frequencies (percentages), with activity in 2019 as the 100% reference. Changes were expressed as percentages: *p* vs. *pp, lp* vs. *pp*. Comparisons in the visit volume were made using the chi-square test. The null hypothesis is that the pandemic had no effect on the volume of outpatient oncology activity; values of *p* < 0.005 were considered statistically significant.

## 3. Results

Results for each center are reported below.

### 3.1. AUSL IRCCS Reggio Emilia (CCCC, COVID mixed, North of Italy)

**Figure 3:**
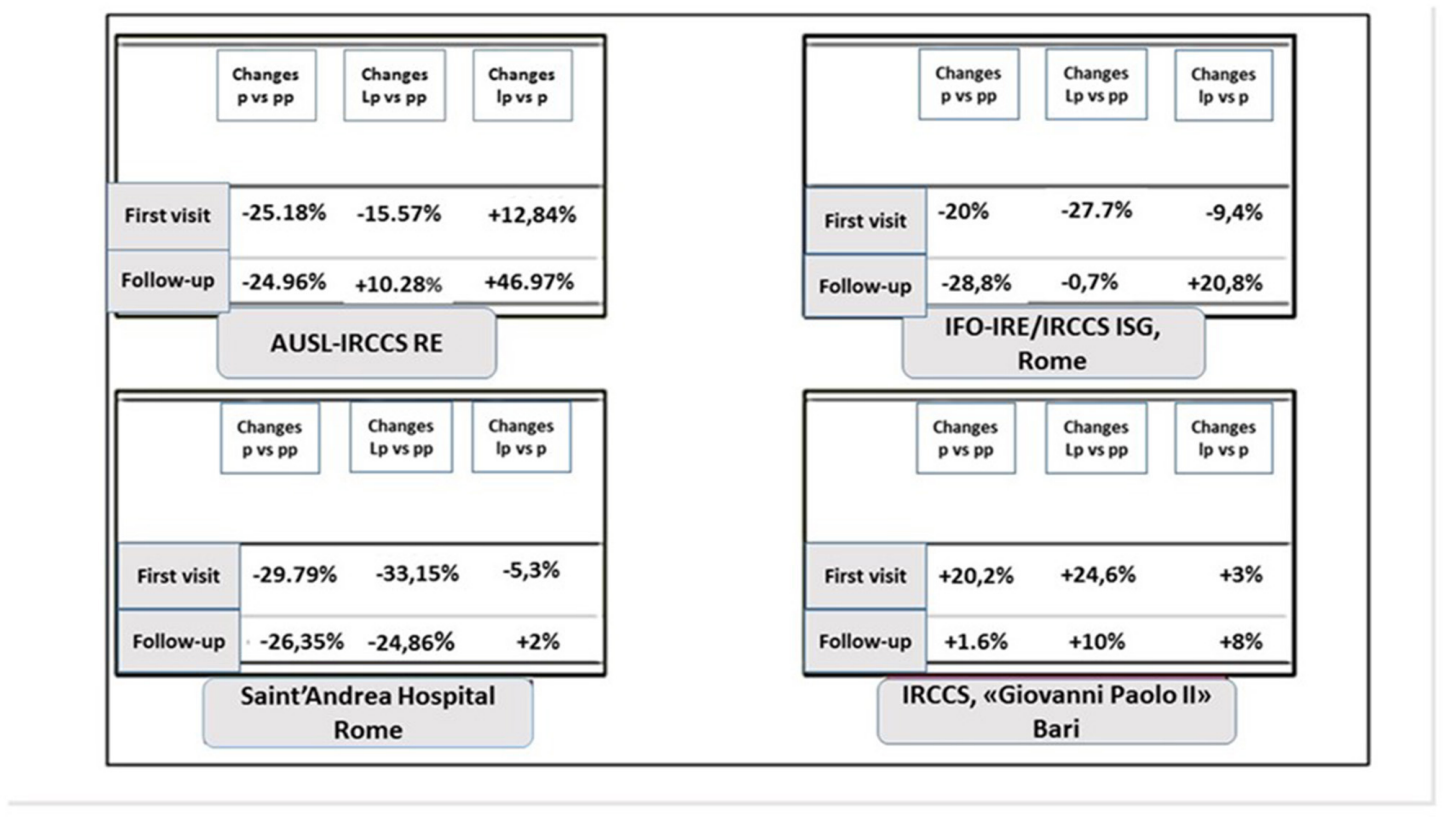
Percentages of visit volume for first appointments and follow-ups of outpatients for each oncological center. p, pandemic year, 2020; pp, pre-pandemic year, 2019; lp, late pandemic 2021.

**Figure 4:**
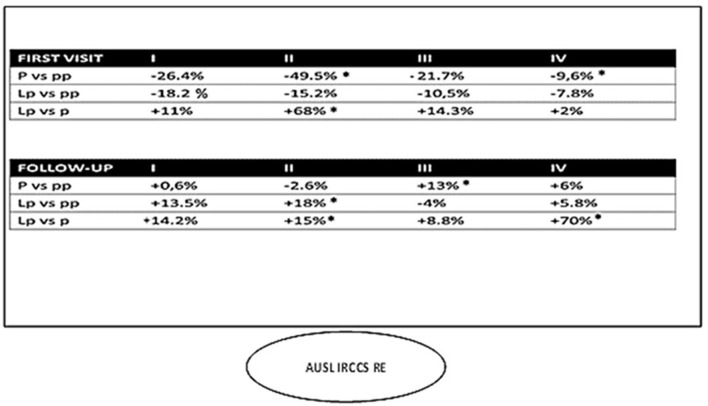
Visit volume at the AUSL IRCCS of Reggio Emilia by quarter. *p < 0.005.

The total volume of first appointments in 2020 and 2021 was down overall (−25.18% and −15.57%, respectively) (Figure 3). When comparing volumes by quarter along the three macro-periods (pre-pandemic, pandemic, and late pandemic), a statistically significant decrease was observed in the second and fourth quarters of 2020 (−49.5 and −9.6%, respectively) (Figure 3). There was no significant decrease in the other quarters (*p* > 0.005). Conversely, the total volume of follow-up visits increased in 2020 and 2021 (+10.28 and +47%, respectively) (Figure 3), with a statistically significant increase of 13% in the third quarter of 2020 (Figure 3). In 2021, the most impressive increase was observed in the fourth quarter of follow up visits (+70%). However, a positive trend was observed throughout the whole *lp* year (Figure 4).

### 3.2. IFO: Istituti fisioterapici ospitalieri/istituto regina elena (IRE)- istituto san gallicano (IRCCS– ISG) (CCCC, COVID –free, Center of Italy)

**Figure 5:**
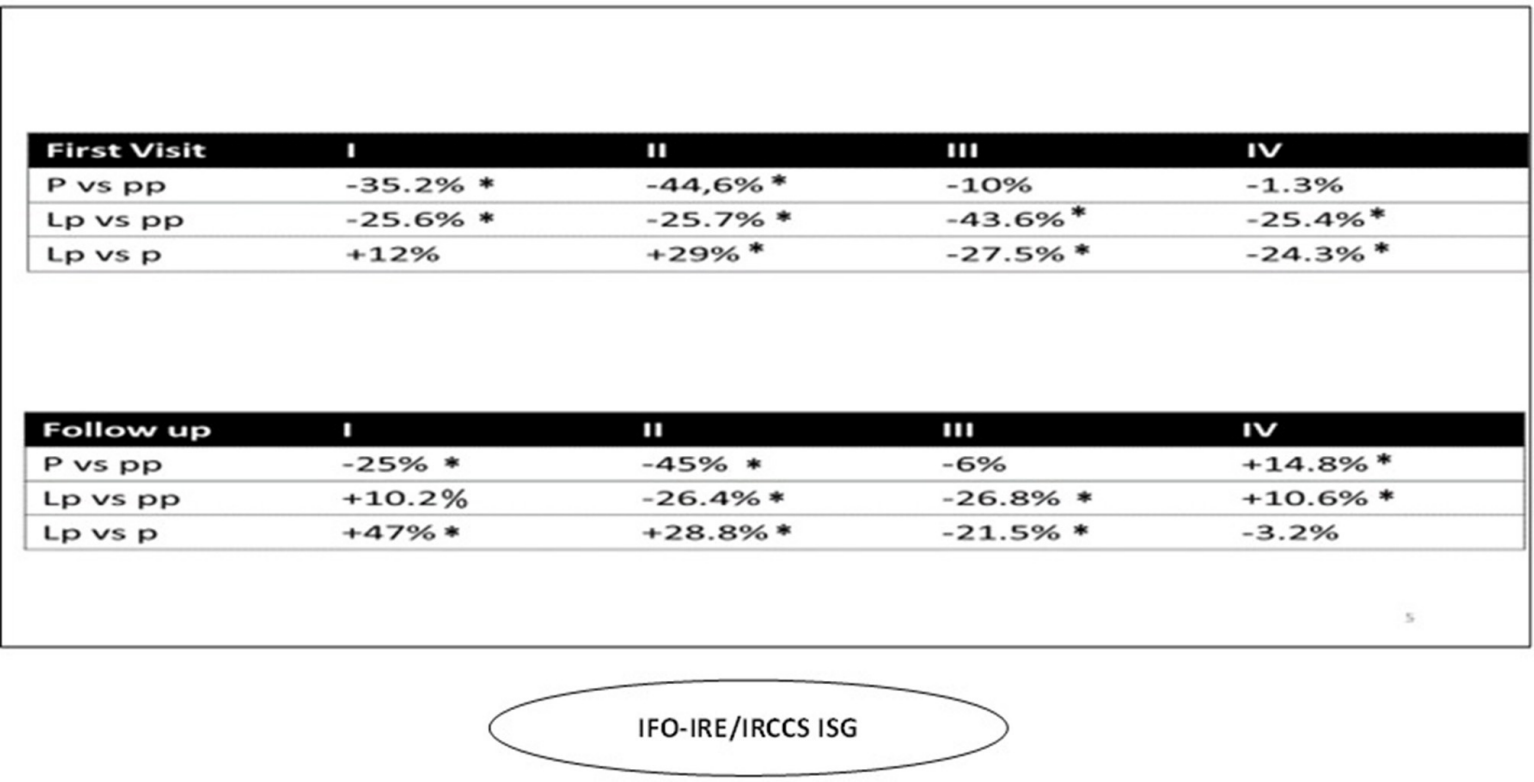
Visit volume at the IRCCS IFO-IRE/IRCCS ISG (Rome) by quarter. *p < 0.005.

The total volume of first visits in 2020 and 2021 was down overall (−20 and −27.7%, respectively) (Figure 3) and did not recover in the *lp* period (−9.4%). The first and second quarters of 2020 compared to the previous year and the third quarter of 2021 were affected by a sharp decline in first visit volume. An upward trend was observed in the third and fourth quarters of 2020, but it was reduced compared to 2019 activity (−10 and −1.3%, respectively). Recovery was not achieved in 2021 (*lp* vs *pp*: −27.5%) (Figure 5). The total volume of follow-up visits in 2020 and 2021 was down overall (−28.8 and −0.7%, respectively) (Figure 3). This KPI showed the same downward trend as that of first visits (Figure 5).

### 3.3. IRCCS “Giovanni Paolo II” of Bari (CCCC, COVID-free, South of Italy)

**Figure 6:**
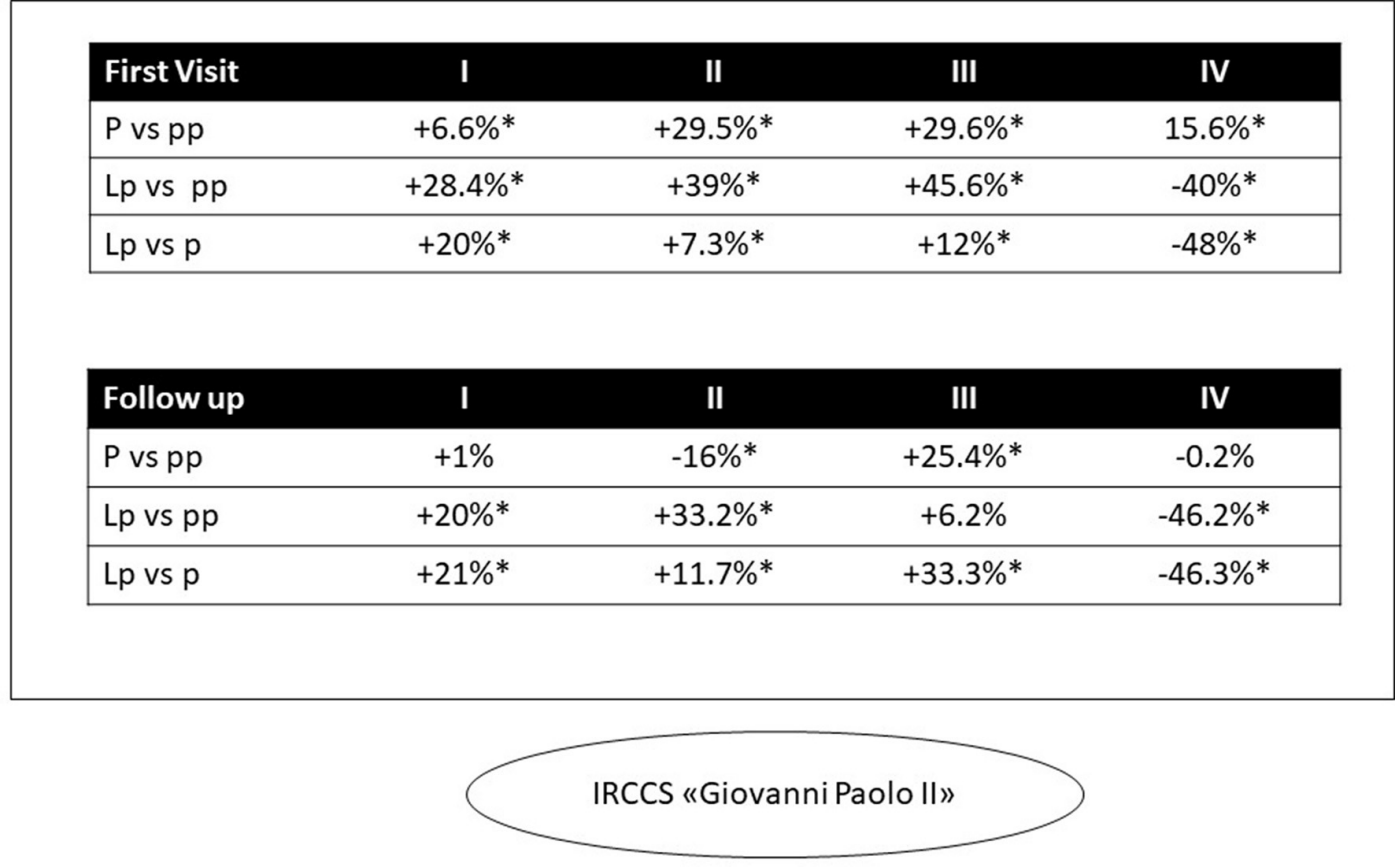
Visit volume at the IRCCS Giovanni Paolo II (Bari) by quarter. *p < 0.005.

Initial outpatient visits increased in 2020 and 2021 (+20.2; +24.6, respectively) (Figure 3). This trend was seen in every quarter in both pandemic years except for a decrease in the fourth quarter of 2021. In the second quarter of 2020, there was a decrease in oncology follow-up visits (-16%) compared with 2019 (Figure 6). Then, in the third quarter of 2020, there was a renewed increase in follow-up appointments, as well as an increase in initial oncology outpatient consultations. In the *lp* period, we observed a more fluctuating trend with a decrease in the second and fourth quarters. For follow-up visits, the same trend was observed with less fluctuation (+1.6 and 10% in the *p* and *lp* periods, respectively) (Figure 3).

### 3.4. Saint'Andrea Community Hospital-University (Community Hospital, Swinging modality, Center of Italy)

**Figure 7:**
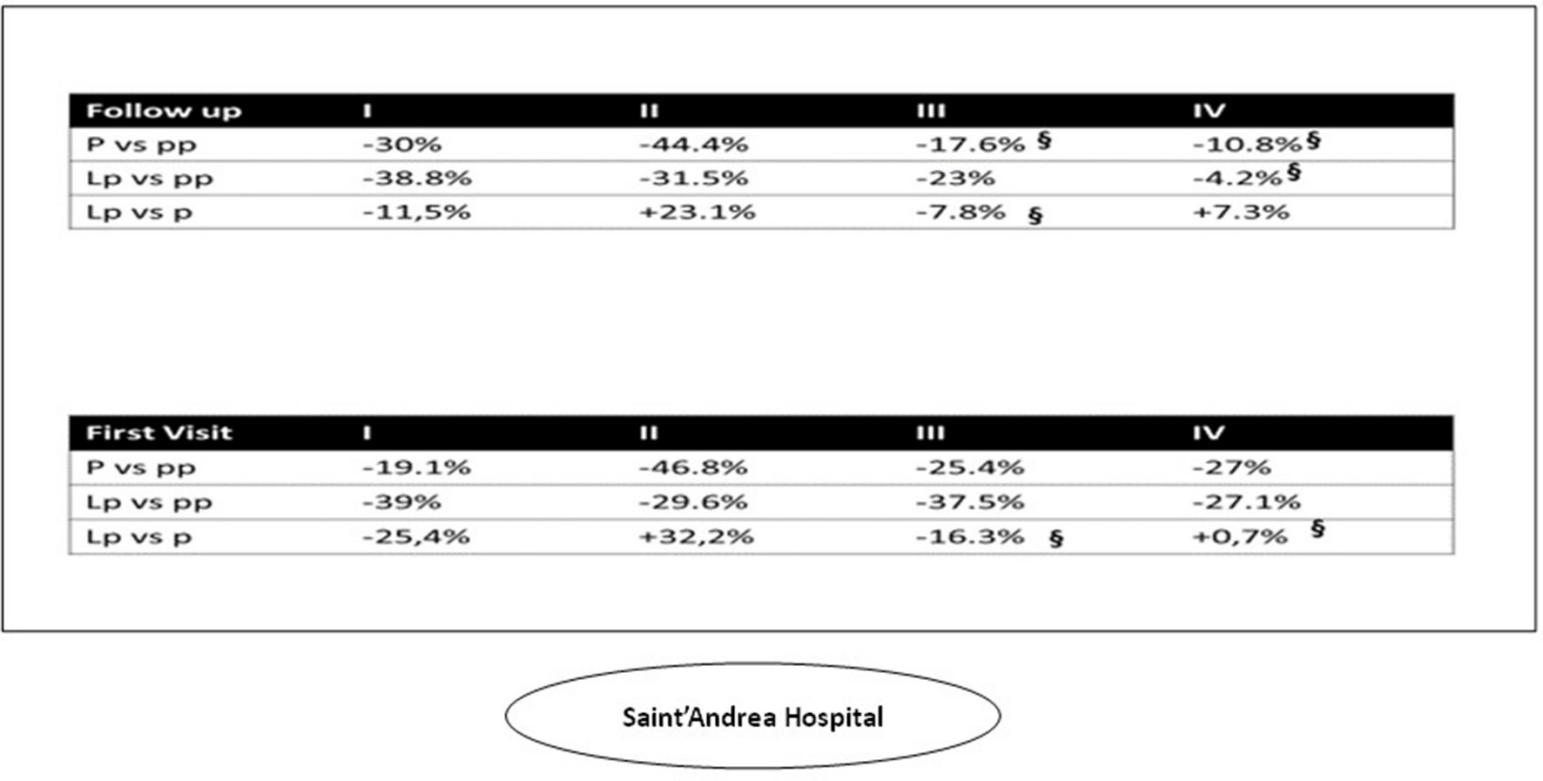
Visit volume at the Saint'Andrea Community Hospital/University (Rome) by quarter. ^§^*p* > 0.005.

The total volume of first visits in 2020 and 2021 was down overall (−29.79 and −33.15%, respectively) (Figure 3). When broken down by quarter, a significant decrease was observed (−30 and 44.4%, respectively; *p*: 0.005). For period *p*, we also estimated a 5.3% decrease in the total volume of first-time visits to *lp* (Figure 3), but this was not statistically significant for the entire year. By comparing data from periods at the same Rt, that is 1.3 (second quarter of 2020 and third quarter of 2021), we found a significant difference of +20% in the volume of first visits (*p* = 0.0004). The total volume of follow-up visits in 2020 was significantly lower (−26.35%) (*p* < 0.005) (Figure 3). When we compared the two periods with higher Rt, a statistically significant increase (+9.07%: *p* < 0.005) in follow-up visits was observed only in the third quarter of 2021 compare to the first COVID-19 wave. Nevertheless, we observed a total 25% decrease in follow-up appointments in 2021 compared to 2019 (Figure 7). When the Rt was expected to rise above 1, this Institute organized its own internal pathways to manage both affected and unaffected patients; this was due to the need to optimize healthcare resources by guaranteeing everyone the minimum standard of assistance in times of increased viral spread. Conversely, when the RT fell below 1, the priority was to ensure the safety of cancer patients. This “swinging” modality started at the beginning of the second COVID-19 wave.

## 4. Discussion

Because the COVID-19 pandemic represents an unprecedented health crisis, epidemiologic data are still needed to develop health policies and strategies for future pandemics of similar scale and complexity (12). This study allowed us to assess the impact of the COVID-19 pandemic on oncology care *via* outpatient “first and follow-up” appointments. Health services are the third pillar in the successful fight against emerging infectious diseases (12) to protect and save lives. In this study, we compared four healthcare facilities in three different Italian regions: three CCCCs [AUSL-IRRCS RE (Northern Italy), IFO (Central Italy), and the IRCCS “Giovanni Paolo II” (Southern Italy)] and an oncology department (Saint' Andrea Hospital) in a Community Hospital affiliated with the Medical University.

At the onset of such a pandemic and in accordance with national and international guidelines for cancer patients, each participating Center reorganized its clinical and nursing services (13).

Furthermore, local recommendations drove the clinical management at each site. AUSL-IRCCS in Reggio Emilia was a COVID-mixed center, while the IFO and IRCCS Giovanni Paolo II followed a COVID-free strategy. Saint'Andrea Hospital adapted its internal organization in a fluctuating or “swinging” modality (i.e., it was alternately a COVID-free and a COVID-mixed hospital according to the Rt).

In 2020, the downward trend seen especially in first appointments of all CCCCs centers, and more so in Saint' Andrea Hospital, was likely related to the onset of visit deferrals as Rt crossed the epidemic threshold (that is “1”). Several reasons could account for these results. First, governments worldwide adopted restrictive measures to contain the spread of the pandemic; in Italy, every region suspended all non-emergency health services (a downward trend in the first period). Second, health professionals were busy caring for hospitalized patients and human resources were drastically cut due to the high number of ill health professionals; therefore, many diagnostic and therapeutic pathways considered “non-urgent” were blocked. In addition, the decrease in first appointments may be due to the population's reluctance to visit hospitals during the COVID-19 outbreak.^3^

Two healthcare facilities (IFO and Saint' Andrea Hospital) operate in the same city (same Rt). Nevertheless, we observed significant differences between these two centers highlighting the impact that internal organizational decisions have on the treatment of vulnerable populations during the pandemic. Notwithstanding a relative improvement when the mixed modality was adopted at IFO (for the first time in the p year, III and IV quarter), at the Saint' Andrea Hospital adopting a “swinging modality” proved to be inefficient in terms of recovery of healthcare services despite an equal Rt factor. Furthermore, in 2021 we observed a minor decrease concerning first and follow-up visit volume compared to *p* year, while only a modest uptrend (+2%) was observed for follow-up visit volume compared to the *pp* year (Figure 3). In fact, adapting the existing space to a new modality by ensuring social distancing in the waiting room most likely led to a reallocation of the reservation agenda to include fewer appointments. Compared to the other CCCCs, the Saint' Andrea Hospital showed substantial differences in visit volume, especially in the *lp* year with respect both to the pandemic and the pre-pandemic period. It is likely that the hospital resources were depleting and remaining resources were reappropriated to manage other critical diseases aside from oncological care, and thus, despite the adoption of mixed modality at the time of increasing viral spread, this strategy did not allow for recovery of outpatient cancer visit volume. It could be hypothesized that the COVID-19 pandemic placed a larger stress on community hospitals, since they do not have the same budget and resources, and have to treat a wider variety of patients, as compared to CCCCs that have a larger budget and focus solely on cancer treatment.

AUSL-IRCCS RE is located in the North of Italy, which was one of the first Italian regions to be affected by the COVID-19 pandemic (Figures 3, **9**). Since the beginning of the COVID-19 pandemic, this Institute decided to prepare multiple safety pathways by separating entrance and exits route with dedicated COVID-19 wards and separate elevators. Having strong COVID-19 measures in place allowed for a prompt recovery post-pandemic, as both safety and services were ensured. Montella et al. (14) had already highlighted the importance of the safety pathway design for healthcare facilities for patients not affected by COVID-19 (14).

**Figure 8:**
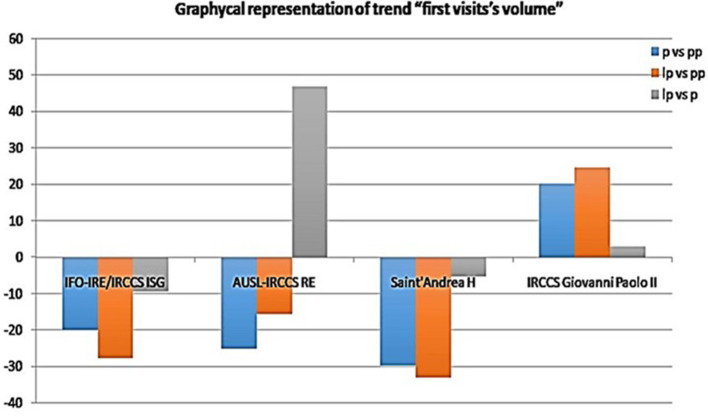
Volume of initial visits over time.

**Figure 9:**
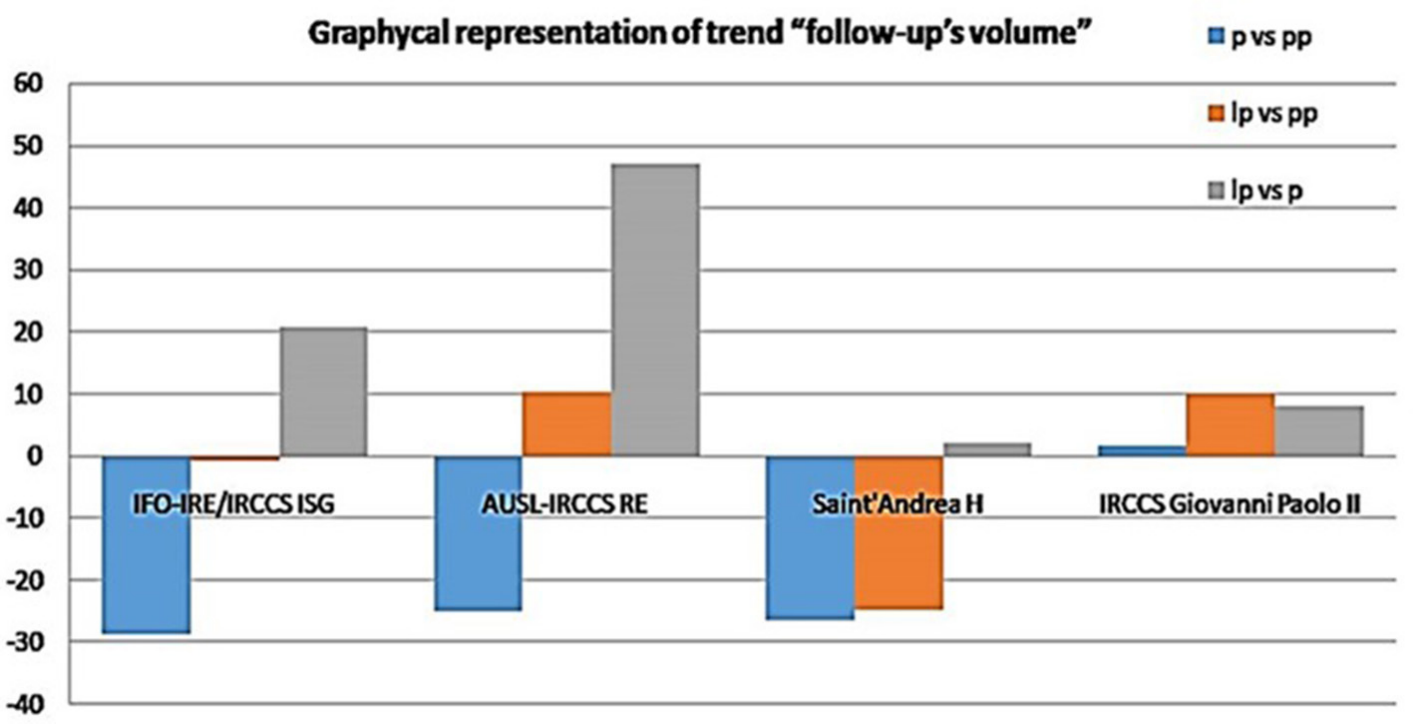
Volume of follow-up visits over time.

With the end of the political restriction in 2020, all visit types gradually resumed. The *lp* period was characterized by an increased volume of first appointments, demonstrating that the *lp* pandemic period was an opportunity for our organizations to face with several issues and try to overcome barriers to care. AUSL-IRCCS RE restored the number of first appointments, although not completely, in 2021. Regarding the follow-ups, IFO and AUSL IRCCS RE resumed their volumes during 2021. On the other hand, Saint' Andrea Hospital did not recover its performance, although it stopped a further downward trend (Figures 8, 9). These results could be related to the different nature of these Institutions. AUSL-IRCCS and IFO are CCCCs, and resources are likely to be well organized for the Institute's tasks, especially for emergencies, compared with oncology services in a Community Hospital. Our results suggest that better organization systems and COVID safety pathways such as dedicated elevators, corridors, waiting rooms, etc), helped Institutions to re-organize healthcare services to accommodate COVID-19 positive patients during the pandemic without compromising the care of cancer patients. Extensive communication between epidemiology departments, health policy makers, and special working groups enabled clinicians to provide services without significant negative impacts on cancer patient management. Our findings underscore the importance of CCCCs that are readily accessible to everyone and can provide follow-up care, considering the increasing prevalence of cancer.^4^

The IRCCS “Giovanni Paolo II” of Bari surprisingly demonstrated an increase in the number of first consultations in outpatient oncology patients (Figure 3), and this healthcare facility warrants an in-depth analysis with further studies to include it in health policies. In analyzing these data, we should take into account that the Puglia region, like several regions of southern Italy, has a passive health mobility compared to northern areas such as Lombardy and Veneto.^5^ In 2017, 58,257 Apulian cancer patients moved outside their region for cancer treatment (see text footnote ^5^). The increasing number of initial outpatient oncology consultations at IRCCS Giovanni Paolo II has led to two hypotheses. First, this result could be due to concerns about mobility toward regions with a high incidence of SARS CoV-2 infection, such as Lombardy and Veneto (15), and the closure of regional borders during the pandemic outbreak. Second, setting up a COVID-free hospital could have provided more safety and confidence for cancer patients seeking medical advice without risking in-hospital contagion. The IRCCS Giovanni Paolo in Bari and the IFO in Rome were both COVID-free cancer centers but they showed a large difference in new patient visits and follow-up visit volume. We can argue that the national restriction to cross the regional and national borders, limited the “medical tourism” phenomenon. Furthermore, the difference in the spread of the virus in the Southern regions compared to Central and Northern Italy, could justify these results. To guarantee a COVID-free status a nasopharyngeal swab was mandatory before hospital entrance. In the case of a positive test, the first or follow-up visits were postponed (we evaluated the volume of clinical appointments, not surgical nor urgent). The low increase in follow-up visits (only 1.6%) could be explained by the national recommendations of deferring any non-urgent visits, so to reserve space in the schedule so to reserve room for patients with new diagnoses and urgent cases.

A more stable performance has been observed for AUSL-IRCCS RE, which ranks sixth among the best Italian hospitals. This result strongly suggest that higher technological progress and better infrastructure could rapidly improve the ability to cope with the pandemic crisis (16) and mitigate the “recession” immediately after the pandemic outbreak (16). A different pathway for the admission of patients with SARS-CoV2 infection to the hospital, with different checkpoints at the entrances of the hospital, has ensured that the oncology care could be carried out without further delays and worries for the patients. In other Institutions, where analyses showed significant variation in follow-up throughout the period, this could be due to a lack of information on the implementation of other modalities to prioritize and ensure follow-up (telemedicine). However, this is beyond the purpose of this study, and consistent data on this topic were not available at the time of publication.

Cancer Core Europe (a network of seven CCCCs across Europe) summarized the actions taken during the first wave of the COVID-19 pandemic. This network highlighted the differences in implementation strategies that resulted from different health care organizations, as well as the urgency of action in countries that were affected differently by the COVID-19 pandemic over time (17). Van de Haar et al. (17) proposed schemes for prioritizing patients for cancer treatment. However, because of the rapidity of change, these are not immediately transferable to the clinical context unless a robust body of knowledge is in place and a clinical task force is established for dynamic up- and down-scaling (17). Even though other studies have examined the impact of COVID-19 on cancer care (18–22), our study is the first step for coordination with other national hospitals.

Our study did not consider demographic characteristics of cancer patients (including citizenship) or clinical outcomes because our goal was to determine the magnitude of the impact of the COVID-19 pandemic on visit volume of cancer outpatients as an indirect assessment of the resilience of our systems to the daunting challenge posed by COVID-19. Zeilinger et al. demonstrated that people with lower socioeconomic educational status were less likely to be seen in outpatient services (23), suggesting an exacerbation of the impact of the COVID-19 pandemic on preexisting inequities in cancer care (24). However, because our national health care system is free and accessible to everyone, socioeconomic and educational status should not affect outcomes.

A Belgian retrospective cohort analysis showed no difference in the total number of outpatient specialist visits compared with a similar period in 2019 (25), whereas we observed a significant decrease with regional differences.

Further consideration could be drawn about the role of vaccination as a plausible factor able to modify some trends, but this is far from the purpose of this study.

As mentioned earlier, our results also shed light on the phenomenon of cross-border health care. Patients use health care services beyond their borders for a variety of reasons. Health tourism includes patients who seek health services in other countries to obtain uninsured services, avoid longer waiting times, or obtain less expensive or perceived better health care. Policy makers around the world are focusing on medical tourism as a welfare and development strategy, and research needs to address it as well (26). There is compelling evidence of how medical tourism can affect public resources and exacerbate health inequities (27, 28). Despite measures to facilitate outbound medical travel from source countries and to build supply-side capacity and competitiveness in destination countries, the systemic drivers of health tourism encompasse health system deficiencies, lack of health care, and regulatory barriers that are not adequately addressed. In our country there is a similar phenomenon across regions and in particular the patient movement from the region of the South toward healthcare facilities located in the richest regions of the country (Emilia Romagna and Lombardy). Before the COVID-19 outbreak, cancer patients made up 80% of the patients, who moved from South to North, doubling the proportion between the Northern and Southern regions. Indeed, if only 4,000 to 6,000 patients living in the North move annually for treatment of oncohematological diseases (generally toward neighboring regions), the figure reaches an astounding 8,000 moving from Sicily, 9,000 from Calabria and as many as 12,000 from Campania to the North.^6^,^7^ As IRCCS of BARI (South of Italy) showed an atypical increase in initial and follow-up visits during the first year of the COVID-19 pandemic, there is a suggestion that this is from patient migration, although further analyses will be necessary. This issue is essential to understand for our national health care system, where the South's economic situation is a long-standing issue and challenge. We believe this pandemic may serve as an opportunity to encourage patients to refer to their regional Institutions and receive treatment locally, but more economic resources will also need to be dedicated to those areas. A more detailed communication about available facilities and quality of care could be a milestone for the oncology task force and the National Minister of Health (29). Regarding medical tourists from abroad, due to national border closures, we did not consider them in term as a factor in this analyses.

This study is the first to analyze the impact of COVID-19 by providing a country-specific picture and looking at three Italian different regions impacted by the COVID-19 outbreak. National guidelines were the same for all regions, but the impact was different. Although Northern Italy was the first and most affected part of the Italian country (at least during the first phase of 2020), our study highlighted probable logistical reasons for the different impact on healthcare facilities rather than epidemic factors.

A limitation of this study might be due to the data sources. Because we analyzed administrative data, the difficulties encountered by clinicians in completing bureaucratic tasks during this period could affect the quality of the data (underestimation); however, because we matched results from the regional registry, epidemiology department and the electronic clinical registry, we are confident that we kept this risk low.

## 5. Conclusions

The COVID-19 pandemic overwhelmed our health care facilities, hurting vulnerable patient populations such as cancer patients. The middle and long-term effects are not well understood. While the delay in access to care associated with the COVID-19 pandemic has received growing attention, raising concerns about the health of the general population in the future, the full range of gaps in cancer care, mainly outpatient cancer services, are not yet well documented. Diagnosis and management of cancer are time-sensitive, and these disruptions could significantly affect it. We recognize the importance of keeping people free to choose where to be cared for; however, there is the need to analyze carefully the reasons why people living in the South of Italy move extensively to the North to seek health care. Perhaps the unfounded belief that better performance in healthcare may only be provided out of one's own region and the absence of adequate health policies are critical issues worthy to be addressed. Many questions remain open. How will the pandemic years affect the mortality of cancer patients due to delay in timely diagnosis and follow-up of therapies? How can policies for a community hospital ensure adequate and timely care for cancer patients and save resources? How can telehealth or other new approaches be valid substitutes for clinical appointments? Further studies are mandatory to clarify the best cancer visit and follow-up options to adopt during an airborne infectious disease health crisis line COVID-19.

## Data availability statement

The raw data supporting the conclusions of this article will be made available by the authors, without undue reservation.

## Author contributions

VF: conceptualization and original draft preparation. VF and MA: methodology. VF and OB: formal analysis. VF, VA, AT, GCa, and AD: data collection. VF and RC: figures and tables draft. All authors contributed to the article and approved the submitted version.

## Footnotes

^1^Available online at: https://www.cancer.gov/publications/dictionaries/cancer-terms/def/comprehensive-cancer-center.

^2^Available online at: https://covid19.infn.it/sommario/rt.htmlx.

^3^ https://www.medicaldevice-network.com/news/people-reluctant-to-visit-hospitals-during-COVID-19/

^4^ https://gco.iarc.fr/today/data/factsheets/populations/380-italy-fact-sheets.pdf

^5^ https://www.sanita.puglia.it/news-in-archivio_det/-/journal_content/56/20182/sanita-aress-su-dati-mobilita-passiva

^6^Available online at: https://soleterre.org/news/la-migrazione-sanitaria-in-italia/.

^7^Available online at: http://www.quadernidellasalute.it/imgs/C_17_pubblicazioni_1004_allegato.pdf.
